# Climate warming will increase chances of hybridization and introgression between two *Takydromus* lizards (Lacertidae)

**DOI:** 10.1002/ece3.7671

**Published:** 2021-05-11

**Authors:** Kun Guo, Jun Zhong, Fan Xie, Lin Zhu, Yan‐Fu Qu, Xiang Ji

**Affiliations:** ^1^ Jiangsu Key Laboratory for Biodiversity and Biotechnology College of Life Sciences Nanjing Normal University Nanjing China; ^2^ College of Life and Environmental Sciences Wenzhou University Wenzhou China

**Keywords:** allopatry, climatic niche, hybridization and introgression, species distribution models, sympatry, *Takydromus* lizards

## Abstract

Coexisting species may experience population and range changes alone or jointly in response to environmental change. Here, we used six climate variables and ten modeling algorithms to predict the distribution of two *Takydromus* species (*T*. *septentrionalis* and *T*. *sexlineatus*) in China. We identified the sympatric and allopatric areas by comparing projections between the two species based on habitat suitability under present and future climate scenarios. We constructed the hypervolumes of six climate variables for the two species and then evaluated overlaps between hypervolumes. From this study, we know the following. First, minimum temperature of coldest month contributes the most to the prediction of habitat suitability. Second, habitats suitable for the two species will shift northward in response to climate warming. Third, the range of *T. sexlineatus* will expand across the four future time intervals before 2,100, namely the 2021–2040, 2041–2060, 2061–2080, and 2081–2100 intervals, under both Shared socioeconomic pathway (SSP) 245 and SSP585 scenarios, and the range of *T. septentrionalis* will also expand in the future except at the 2081–2100 interval under the SSP585 scenario. Fourth, the sympatric areas will contract or expand under the SSP245 scenario and expand across the four future time intervals before 2,100 under the SSP585 scenario. Fifth, the niche hypervolumes of the two species partially overlapped, and the differences in niche centroid show some degree of niche differentiation between the two species. These results allow to conclude that climate warming will not only drive the northward drift of sympatric areas but also increase the size of these areas if nothing is done to limit the emission of greenhouse gases. Given the existence of hybridization and introgression between *T. septentrionalis* and *T. sexlineatus* in the field where they coexist, we also conclude that climate warming will increase chances of hybridization and introgression between the two species.

## Graphical abstract

Climate warming will not only drive the northward drift of sympatric areas but also increase the size of these areas if nothing is done to limit the emission of greenhouse gases in the future. Given the existence of hybridization and introgression between two Takydromus lizards in the field where they coexist, climate warming will increase hybridization and introgression between the two species.

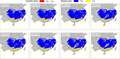

## 1 INTRODUCTION

Speciation is the origin of reproductive isolation and divergence between populations (Mayr, 1963). Isolation by physical and/or ecological barriers is an important driver in allopatric processes that may lead to population differentiation and, ultimately, speciation (Volis et al., 2019). Other processes such as hybridization and introgression between involved species in the secondary contact zones may also have important evolutionary consequences for speciation, especially at the early stages of speciation when reproductive barriers may be weak (Bertrand et al., 2017; Cortés‐Ortiz et al., 2019; Coyner et al., 2015; Sardell & Uy, 2016; Souissi et al., 2018). There has been evidence that gene flow re‐established by hybridization and introgression may lead to genetic re‐admixture between closely related species living in sympatry (Mapel et al., 2021; Martin et al., 2015; Seehausen, 2006). Environmental change as one of the most serious challenging concerns poses multiple threats to organisms, results in shifts in species distribution, and alters interspecific interactions by breaking the physical (geographical) barriers of reproductive isolation and thus the chances of hybridization and introgression (Bellard et al., 2012; Mammola et al., 2019; Parmesan, 2006; Zhang, Mammola, et al., 2020).

Coexisting species may experience population (expansions or declines) and range (expansions or contractions) changes alone or jointly in response to environmental change. Climate warming is one hypothesis often proposed to explain these changes (Liu, White, et al., 2011; Liu, Guo, et al., 2011; Sinervo et al., 2010; Zhang, Capinha, et al., 2020). The earth's temperature has increased by ~0.7°C over the past century, which may substantially alter the entire ecosystem and constitute a serious threat to global biodiversity (McCarthy et al., 2010). In lizards, for example, such a warming trend will lead 39% of global populations and 20% of global species to go extinct by 2080, under the premise of a limited potential for plastic and/or genetic adjustments in response to a warming world (Sinervo et al., 2010). Therefore, it is of great importance to evaluate whether and how climate warming alters the distribution of habitats suitable for coexisting species.

Species distribution models (SDMs) are a powerful tool for delineating habitat suitability in geographic space by statistically exploring the relationships between species occurrence records and environmental variables (Elith & Leathwick, 2009; Guisan & Thuiller, 2005; Guisan et al., 2017; Guisan & Zimmermann, 2000; Phillips et al., 2006). SDMs have been widely used to examine the effects of environmental change in species distribution and their interactions with other species (Austin & Van Niel, 2011; Guisan et al., 2013; Vaclavik & Meentemeyer, 2012), with consensus methods found to be able to cope with prediction variability by combining an ensemble of predictions from different modeling methods (Araújo & New, 2007). The ensemble SDMs simultaneously use several algorithms to predict the present and future maps of habitat suitability, thus avoiding the limitation of occurrence records in data analysis and, compared with single algorithms, improving the accuracy of model predictions (Araújo & New, 2007). Moreover, the projection of ensemble SDMs will help to find the previously undetected distribution of species. Using ensemble SDMs, one may not only determine sympatric and allopatric areas of coexisting species in different periods but also better explain discordance of genome or gene introgression between species in secondary contact zones (Zhang, Capinha, et al., 2020).

Asian grass lizards of the genus *Takydromus* (Lacertidae) are widely distributed in East and Southeast Asia, southward to northeastern India, Borneo, Sumatra, Bangka, and Java, and northward to the Russian Far East (Arnold, 1997; Arnold et al., 2007). Of the 24 currently recognized species, 15 occur in China, eight only in mainland China, six only in Taiwan, and one (*T. kuehnei*) on both sides of the Taiwan Strait (Wang et al., 2019). Widespread sympatry between different pairs of *Takydromus* species occurs in several areas of mainland China, such as *T*. *septentrionalis* and *T*. *sexlineatus* in southeastern China, *T*. *intermedius* and *T*. *septentrionalis* in southwestern China, *T. kuehnei* and *T*. *sexlineatus* in southern China, *T*. *septentrionalis* and *T*. *wolteri* in eastern China, and *T*. *amurensis* and *T*. *wolteri* in northeastern China (Liu, 1999). Over the past decade, four new *Takydromus* species have been described in China, two [*T. albomaculosus* (Wang et al., 2017) and *T. yunkaiensis* (Wang et al., 2019)] in mainland China, and two (*T. luyeanus* and *T. viridipunctatus*; Lue & Lin, 2008) in Taiwan. Interestingly, all these newly described species have a narrow range, inferring that processes other than isolation by physical or ecological barriers might lead to speciation. Here, we use an ensemble modeling approach to predict the contemporary and future sympatric and allopatric areas of *T*. *septentrionalis* and *T*. *sexlineatus* in the context of global climate warming. More specifically, we aim to (1) identify climate variables that have a key role in determining the distribution of the two species, (2) predict spatiotemporal variation in sympatric and allopatric areas of the two species, and (3) explore the influence of climate warming on hybridization and introgression between the two species. Given the existence of hybridization and introgression between these two *Takydromus* species in the field where they coexist (Guo, 2019), we predict that these processes of gene exchange will be affected by climate warming if it alters the size of the sympatric areas.

## 2 MATERIALS AND METHODS

### 2.1 Study species

**FIGURE 1:**
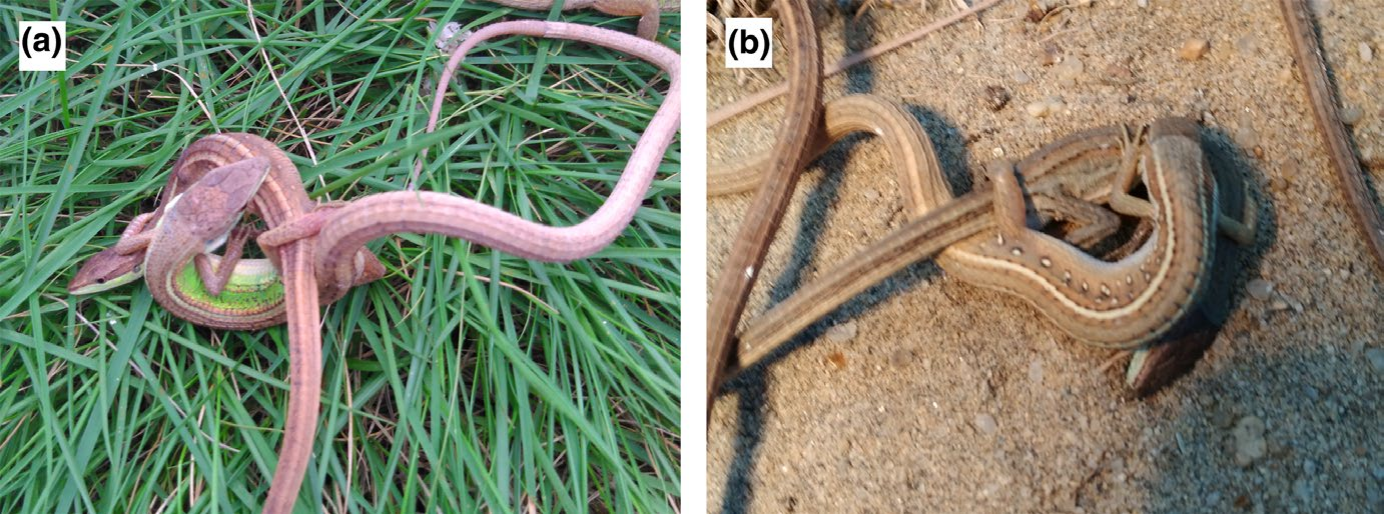
Heterospecific heterosexual mating partners between a male northern grass lizard and a female southern grass lizard (a) and between a female northern grass lizard and a male southern grass lizard (b)

The northern grass lizard (*T*. *septentrionalis*) and the southern grass lizard (*T*. *sexlineatus*) both are small, oviparous, and heliothermic lacertid lizards, with the former endemic to China and more northerly distributed than the latter that is widely distributed in southern China, India (Assam) through Burma and Thailand to Vietnam, south to Sumatra, Java, and Borneo in Indonesia (Liu, 1999). *Takydromus septentrionalis* is a typical temperate lizard with a distributional range that has a temperate and monsoonal climate with four distinct seasons; *T*. *sexlineatus* is basically a warm‐climate lizard with a distributional range that has a subtropical and tropical monsoon climate (Xu & Ji, 2000; Zhang & Ji, 2004). Sympatry between the two species occurs in southeastern China, where they coexist not because they differ so much in the niche axes of diet, space, and time but because they use slightly different microhabitats (Liu, 1999). Female *T*. *septentrionalis* can lay up to 9 clutches with 1–5 eggs each per breeding season from April to August (Ji et al., 2007; Luo et al., 2010); female *T*. *sexlineatus* can lay up to 5 clutches with 1–4 eggs each per breeding season also from April to August (Xu & Ji, 2000; Zhang & Ji, 2004). Evidence from *T*. *septentrionalis* has shown that males are the less choosy sex and can mate at extreme phylogenetic distances, either with conspecific females from distant populations, or even with heterospecific but congeneric females (Guo et al., 2020). Actually, bidirectional heterospecific matings (male *T*. *septentrionalis* × female *T*. *sexlineatus*, or the opposite direction) happen not frequently but often enough in the field and laboratory (Figure 1).

### 2.2 Species occurrence data and climatic variables

Our study area covers the entire range of the two species in southeastern and central China (~17–38°N, ~97–125°E), including localities occupied only by *T*. *septentrionalis*, only by *T*. *sexlineatus*, and by both species (Appendix S1). Occurrence records were from our own field investigation, published papers (Du et al., 2005; Xu & Ji, 2000; Yang et al., 2008) and scientific books (Liu, 1999; Zhao & Adler, 1993), and online datasets such as Global Biodiversity Information Facility (http://www.gbif.org), HerpNet/VertNet (http://www.vertnet.org), and National Specimen Information Infrastructure (http://www.nsii.org.cn). We only used one record in a high‐resolution (2.5 × 2.5 arcmin) grid cell, thereby avoiding overrepresentation of climate data in intensively sampled areas. Of the 156 occurrence records, 127 were from *T. septentrionalis* and 29 from *T. sexlineatus* (Appendix S1).

As has been reported for other taxa, a number of environmental variables can be used to predict the distribution of reptiles (Franklin et al., 2009; Lin & Wiens, 2017; Falaschi et al., 2019; Lin, Chen et al., 2019). Here, we used six climate variables to predict the distribution of the two *Takydromus* lizards, including Bio3 (isothermality), Bio5 (maximum temperature of warmest month), Bio6 (minimum temperature of coldest month), Bio15 (precipitation seasonality), Bio17 (precipitation of driest quarter), and Bio18 (precipitation of warmest quarter). We used these six variables for two reasons. First, they are all nonredundant variables with an absolute value of Pearson's correlation coefficient <0.70 (Dormann et al., 2013); all other unused climate variables had an absolute value of Pearson's correlation coefficient ≥0.70 (Figure S1). Second, they have a comparatively more direct role in influencing the life history of lizards (Du et al., 2005; Gao et al., 2014; Lu et al., 2014; Ma et al., 2019). We extracted climate variables for each occurrence point at a spatial resolution of 2.5 × 2.5 arcmin from the WorldClim database (http://www.worldclim.org), using data from 1970 to 2000 as a baseline for the current period. We used the same way to extract climate variables for four future time intervals, namely the 2021–2040, 2041–2060, 2061–2080, and 2081–2100 intervals. The future climate projections were derived from six global circulation models (GCMs): BBC‐CSM2‐MR, CanESM5, CNRM‐CM6‐1, CNRM‐ESM2‐1, MIROC6, and MIROC‐ES2L. Two shared socioeconomic pathways (SSPs), SSP245 (an average scenario) and SSP585 (a pessimistic scenario), were selected to drive climate models for Coupled Model Intercomparison Project Phase 6 (CMIP6). To reduce uncertainties among different GCMs, we averaged projections of the six GCMs as future climates to predict future habitats suitable for the two *Takydromus* lizards.

### 2.3 SDM development for habitat suitability

We used 10 modeling algorithms (see Table 1 for names and abbreviations) suitable for species distribution prediction to correlate the distribution of the studying species with the associated climatic data by the biomod2 (Thuiller et al., 2019) package in R 3.5.1. For algorithms requiring species absence records, we generated 10,000 pseudo‐absence records for the species by randomly selecting these pseudo‐absence records from across the study area (Barbet‐Massin et al., 2012; Elith & Leathwick, 2009; Guisan et al., 2017; Thuiller et al., 2019). All modeling procedures were performed under default settings of the biomod2 package (Thuiller et al., 2019).

**TABLE 1:** Predictive performance based on the TSS and AUC values of ten modeling algorithms used in this study

Algorithms	*Takydromus septentrionalis*		*Takydromus sexlineatus*	
	TSS	AUC	TSS	AUC
Artificial neural network, ANN	0.716 ± 0.010	0.885 ± 0.006	0.827 ± 0.032	0.922 ± 0.013
Classification tree analysis, CTA	0.714 ± 0.026	0.864 ± 0.012	0.851 ± 0.018	0.926 ± 0.009
Flexible discriminant analysis, FDA	0.725 ± 0.018	0.893 ± 0.008	0.916 ± 0.007	0.959 ± 0.004
Generalized additive model, GAM	0.770 ± 0.017	0.900 ± 0.007	0.582 ± 0.058	0.788 ± 0.030
Generalized boosting model, GBM*	0.797 ± 0.015	0.921 ± 0.007	0.822 ± 0.033	0.920 ± 0.031
Generalized linear model, GLM*	0.781 ± 0.015	0.915 ± 0.004	0.860 ± 0.017	0.931 ± 0.005
Maximum entropy, MaxEnt	0.746 ± 0.020	0.894 ± 0.011	0.894 ± 0.010	0.954 ± 0.003
Multiple adaptive regression splines, MARS	0.790 ± 0.012	0.912 ± 0.005	NA	NA
Random forest, RF	0.715 ± 0.010	0.908 ± 0.004	0.840 ± 0.022	0.926 ± 0.013
Surface range envelop, SRE	0.600 ± 0.036	0.800 ± 0.018	0.584 ± 0.059	0.792 ± 0.030

Note

Algorithms GBM and GLM marked with asterisks (*) had good predictive performance and were therefore selected to construct the ensemble model. NA (missing data) indicates algorithms that failed to converge. Data are expressed as mean ± standard error.

We performed a fivefold cross‐validation approach with 10 repetitions to evaluate the predictive performance of each algorithm. We randomly divided the occurrence records into five equal groups (four for constructing prediction models and one for testing the prediction models) and repeated this operation 10 times (Thuiller et al., 2019). The model accuracy in each algorithm was assessed using the true skill statistics (TSS; Allouche et al., 2006) and the area under the receiver operating characteristic curve (AUC; Liu, White, et al., 2011; Liu, Guo, et al., 2011). The algorithms with a mean TSS < 0.75 (Nüchel et al., 2018) or a mean AUC < 0.90 (Araújo et al., 2005) were discarded in subsequent analyses. We used the same algorithms to develop the ensemble SDMs for the two *Takydromus* lizards and compare their habitat suitability under identical conditions (Grenouillet et al., 2011).

We used a randomization methodology in the selected algorithms to assess the relative importance of climate variables used to predict species habitat suitability (Stanton et al., 2012), and then calculated the response curve of the most important variable affecting the polarization distribution of the *Takydromus* lizards. Both occurrence and pseudo‐absence data were used to predict habitat suitability under the present and future climatic conditions. We got the final result with maps to show habitats suitable for *T. septentrionalis* and *T. sexlineatus* by the committee averaging of predictions of the selected model algorithms, and then converted the model outcomes into binary (suitable or unsuitable) maps by a threshold of the maximizing TSS (Liu et al., 2013). We identified the sympatric and allopatric areas by comparing projections between *T. septentrionalis* and *T. sexlineatus* based on their habitat suitability under present and future climate change scenarios. Habitats in sympatric areas are suitable for both species, and habitats in allopatric areas are suitable only for one species.

### 2.4 Comparison of exploited climatic niche

To compare the exploited climatic niche between the two *Takydromus* lizards (i.e., specialization or generalization), we used the hypervolume package in R 3.5.1 to estimate the climatic niche of each species using a stochastic geometry approach (Blonder et al., 2014, 2018). We constructed the hypervolumes of six climate variables for the two species, which are the definitiveness of habitat suitability and have been used in SDMs. In addition, hypervolumes were randomly resampled to yield 3,000 points across all datasets for each species, thereby adjusting for the influence of sample size on hypervolumes and overlaps. After hypervolume construction, we calculated the Euclidean distance, the Sørensen and Jaccard similarity index, and the unique volume fraction to evaluate overlaps between hypervolumes (Helsen et al., 2020).

## 3 RESULTS

### 3.1 Model performance

TSS and AUC values for the 10 modeling algorithms showed high levels of predictive performance in both species; mean TSS values varied from 0.600 to 0.797 in *T. septentrionalis* and from 0.582 to 0.916 in *T. sexlineatus*, and mean AUC values varied from 0.800 to 0.921 in *T. septentrionalis* and from 0.788 to 0.959 in *T. sexlineatus* (Table 1). Comparatively, GBM and GLM performed better than the other eight algorithms in constructing the ensemble modeling for the two species.

### 3.2 Variable contribution and response curves

**FIGURE 2:**
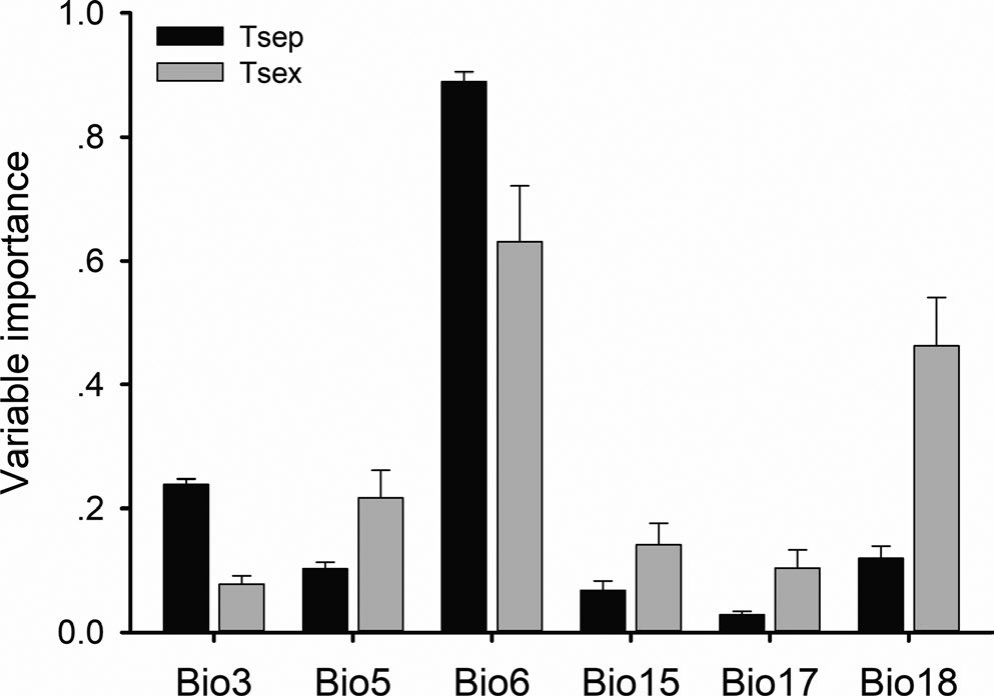
The relative contributions of the six climatic variables used in this study to the species distribution models for *T. septentrionalis* (Tsep) and *T. sexlineatus* (Tsex). Data are expressed as mean ± standard error

**FIGURE 3:**
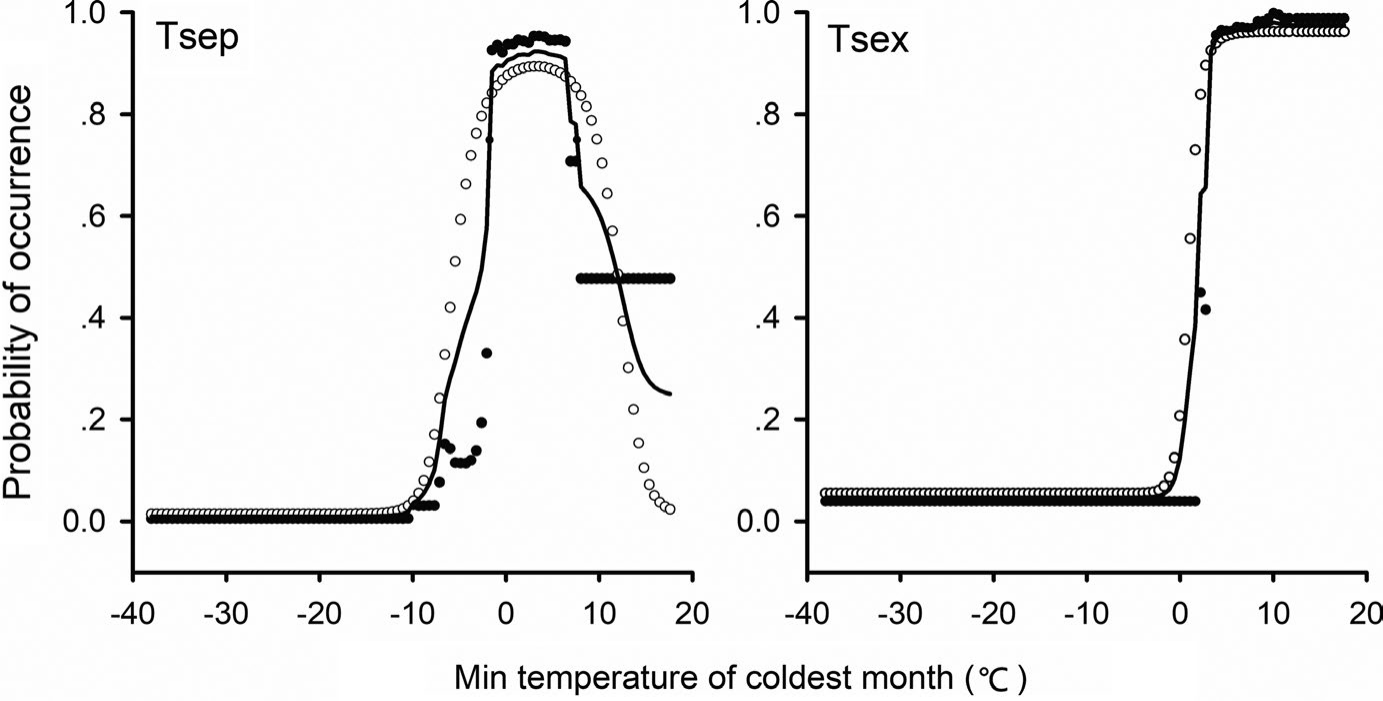
Response curves of the predicted occurrence probability of *T. septentrionalis* (Tsep) and *T. sexlineatus* (Tsex) against Bio6 (minimum temperature of coldest month, °C). Solid circles depict response curves of the average predicted occurrence probability in GBM approached with 10 repetitions, hollow circles depict response curves of the average predicted occurrence probability in GLM approached with 10 repetitions, and lines depict response curves of the average predicted occurrence probability in two algorithms

Among the six climate variables, Bio6 contributed the most to the prediction of habitat suitability in both species, with its relative contribution accounting for approximately 89% in *T. septentrionalis* and 63% in *T. sexlineatus* (Figure 2). The response curves of the predicted occurrence probability against Bio6 varied with algorithms and species in the projections. *Takydromus septentrionalis* was predicted to have a high probability of occurrence in regions with Bio6 varying from −1.0 to 6.2°C, and *T. sexlineatus* in regions with Bio6 greater than 3.9°C (Figure 3).

### 3.3 Present and future projections of habitat suitability

**FIGURE 4:**
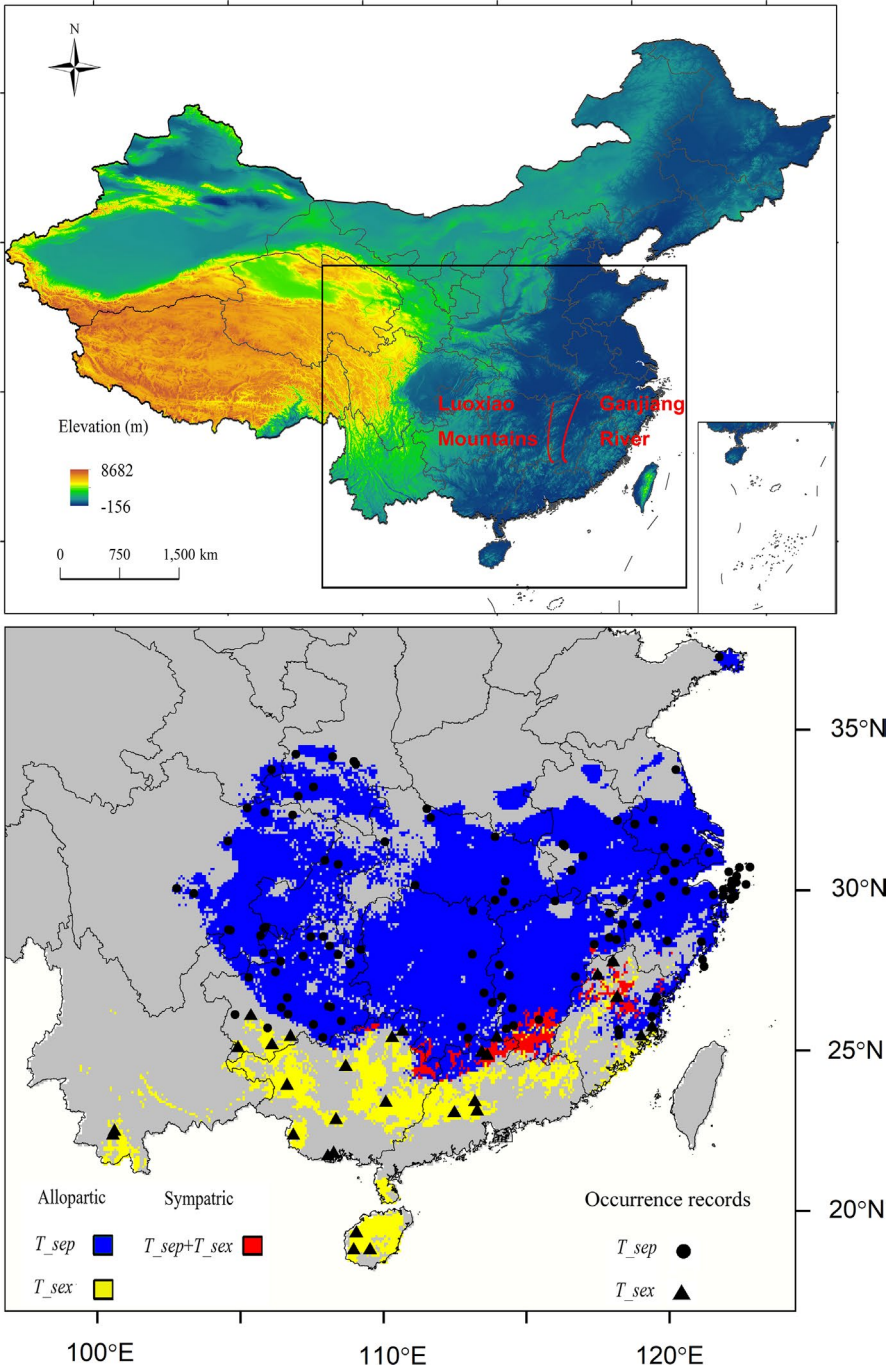
The predicted current sympatric and allopatric areas of *T. septentrionalis* (Tsep) and *T. sexlineatus* (Tsex). The available occurrence records of Tsep (black dots) and Tsex (black triangles) were used to build the species distribution models for the two species. The maps show the areas sampled in context with surrounding landmasses

From present SDM projections (Figure 4), we know the following. First, habitats suitable for *T. septentrionalis* are mainly located in the central and southeastern provinces (Anhui, Fujian, Guangdong, Guangxi, Guizhou, Henan, Hubei, Hunan, Jiangxi, Jiangsu, Shaanxi, Sichuan, Shandong, Yunnan, and Zhejiang) or province‐level municipalities (Chongqing and Shanghai) of China. Second, habitats suitable for *T. sexlineatus* are mainly located in the southern provinces (Fujian, Guangdong, Guangxi, Guizhou, Hainan, and Yunnan) of China. Third, sympatry between the two species occurs in Fujian, Guangxi, Guangdong, Guizhou, Hunan, and Jiangxi provinces (Figure 4).

**FIGURE 5:**
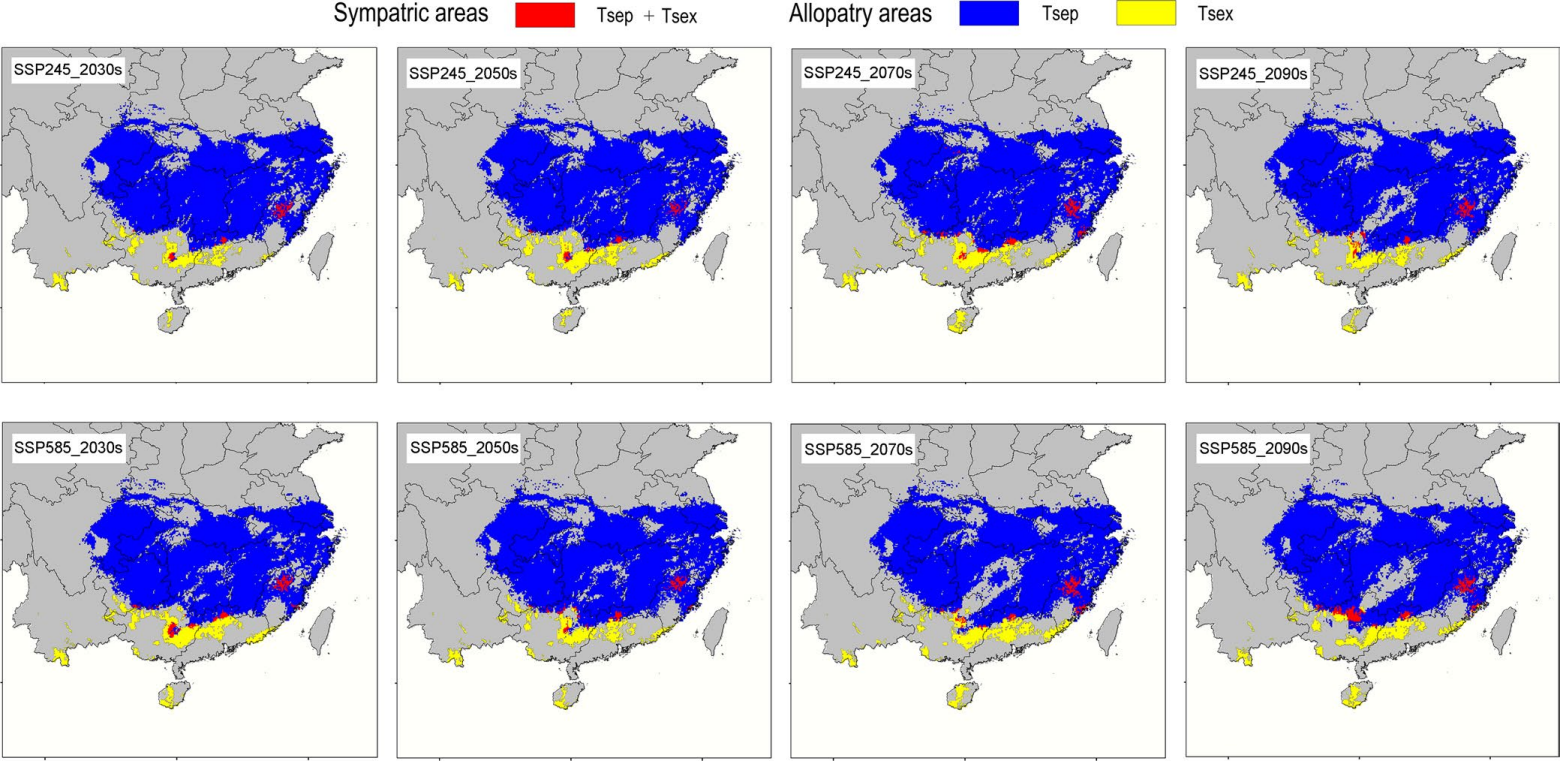
Temporal shifts in the sympatric area of *T. septentrionalis* (Tsep) and *T. sexlineatus* (Tsex) under two scenarios at the 2021–2040, 2041–2060, 2061–2080, and 2081–2100 time intervals using averages of six global circulation models as future climates. The maps show the areas sampled in context with surrounding landmasses

Habitats suitable for the two *Takydromus* species will shift in the future in response to climate warming, with changes in range size varying with the SSPs using averages of six GCMs as future climatic conditions (Figure 5). The range of *T. sexlineatus* will expand across the four future time intervals before 2,100 under both SSP245 and SSP585 scenarios (Table 2). The range of *T. septentrionalis* will also expand in the future except at the 2081–2100 interval under the SSP585 scenario (Table 2). Habitats suitable for the two species will both shift northward in the future, although they respond differently to future climates. Habitats suitable for *T. septentrionalis* will fade away in Shandong province and decline to some extent in the middle of the species’ range, whereas habitats suitable for *T. sexlineatus* will expand in Guangxi, Guizhou, and Yunnan provinces (Figure 5). The sympatric areas of the two species will either contract or expand in the future under the SSP245 scenario and expand across the four future time intervals before 2,100 under the SSP585 scenario (Table 2); all of these sympatric areas will be still in Fujian, Guangdong, Guangxi, and Guizhou provinces (Figure 5). More specifically, the sympatric areas will contract at the 2021–2041, 2041–2060, and 2081–2100 intervals but expand at the 2061–2080 interval under the SSP245 scenario, and expand across the four time intervals under the SSP585 scenario (Table 2). Only one sympatric area surrounded by habitats of *T. septentrionalis* in Wuyi Mountain of Fujian province will be persistent over time (Figure 5).

**TABLE 2:** Percentage changes in sympatric and entire distributional ranges of two *Takydromus* species under future climate scenarios at the 2021–2040, 2041–2060, 2061–2080, and 2081–2100 time intervals as estimated by the ensemble species distribution model using averages of six global circulation models as future climates

Time interval	Sympatric distribution		*Takydromus septentrionalis*		*Takydromus sexlineatus*	
	SSP245	SSP585	SSP245	SSP585	SSP245	SSP585
2021–2040	−40.6	6.0	17.0	18.4	24.2	3.5
2041–2060	−35.9	14.4	19.6	17.1	21.7	22.3
2061–2080	16.0	2.5	19.1	3.5	37.0	36.1
2081–2100	−13.2	57.6	13.1	−15.0	19.7	16.0

### 3.4 Overlap of exploited climate niche

**FIGURE 6:**
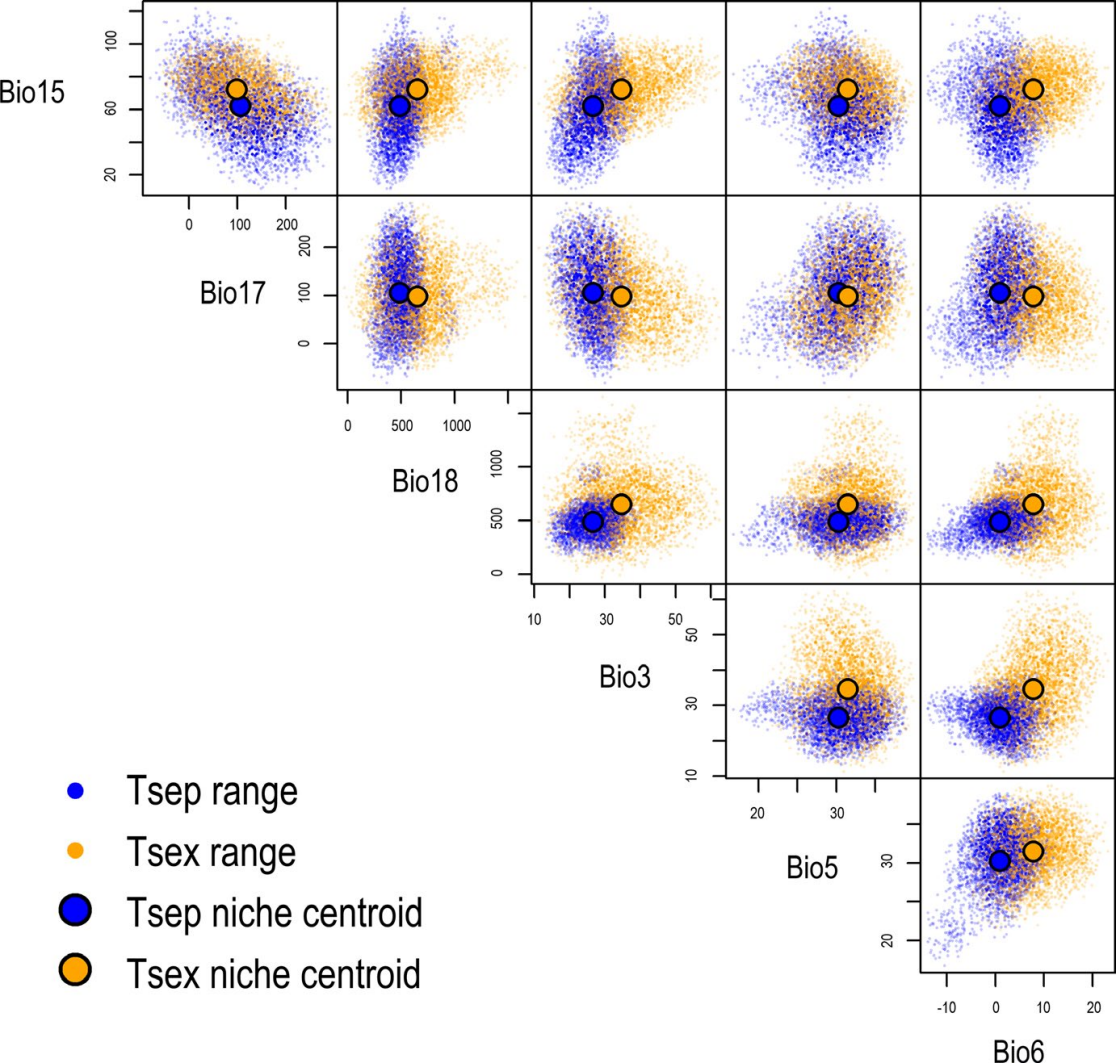
Visualization of the climate niche hypervolumes based on the six climatic variables for the *T. septentrionalis* (Tsep) and *T. sexlineatus* (Tsex). Large dots are hypervolume centroids, and small dots are randomized points sampled from the inferred hypervolume to visualize the stochastic description of each hypervolume

The niche hypervolumes of the two species estimated by six climate variables partially overlapped (Figure 6). The niche hypervolume was much greater in *T. sexlineatus* (8.017 × 10^10^) than in *T. septentrionalis* (1.754 × 10^10^), with an overlapping volume of 1.202 × 10^10^. The Sørensen and Jaccard similarity index of the hypervolume was 0.246 in *T. septentrionalis* and 0.140 in *T. sexlineatus*, suggesting a high degree of niche overlap between the two species (Figure 6). The hypervolume centroids of Bio3, Bio5, Bio6, Bio15, Bio17, and Bio18 in order were 26.7, 30.3, 0.9, 62.1, 105.7, and 489.4 for *T. septentrionalis*, and 34.5, 31.5, 7.9, 72.2, 100.6, and 655.1 for *T. sexlineatus*. These differences in niche centroid provided evidence for some degree of niche differentiation between the two species (Figure 6).

## 4 DISCUSSION

By the end of the 21st century, the global average surface temperature is expected to increase by 3.7°C under the high emission pathway of RCP 8.5 (IPCC Working Group II, 2014). As ambient temperatures rise, many organisms will be subjected to increased selection pressure and even face the risk of local extinction (Sinervo et al., 2010). One way by which organisms may use to cope with the adverse impacts of global warming on their fitness and population persistence is to shift the distributional range and/or the center of distribution (Gienapp et al., 2008; RodríGuez‐Robles et al., 2010). Actually, northward migration under a warming climate has been reported for a diverse array of organism, including plants (Alsos et al., 2012), invertebrates (Bradshaw & Holzapfel, 2006), and vertebrates (Duan et al., 2016; Gienapp et al., 2008; RodríGuez‐Robles et al., 2010). SDM studies generally suggest the distribution of organisms is environmentally dependent (Austin & Van Niel, 2011; Guisan et al., 2013; Vaclavik & Meentemeyer, 2012). However, there is no single algorithm or climate variable that can be used to predict the distribution of all species (Ducci et al., 2015; Qiao et al., 2015). Modeling with a single algorithm may either overestimate or underestimate habitats suitable for a species (Pearson et al., 2006), and using less appropriate climate variables may increase uncertainty in range predictions (Braunisch et al., 2013). Nonetheless, using an ensemble modeling approach and choosing climate variables that directly affect life history may substantially improve the predictive performance of a model and are therefore helpful to obtain a more accurate prediction of habitat suitability for a study species (Thuiller et al., 2009). Here, we used an ensemble of SDMs with high TSS and AUC values to predict habitats suitable for the two *Takydromus* species under the present climate condition. We found that (1) Bio6 had a key role in determining the distribution of the two species; (2) the 156 occurrence records (127 for *T. septentrionalis* and 29 for *T. sexlineatus*) were all in the predicted range of each species; and (3) most records of occurrence were in the range reported for each species (Zhao & Adler, 1993). More interestingly, the model predicted new localities in Wuyi Mountain of Fujian, central Guangxi, and southern Guizhou (Figure 4), where the two species are sympatric, but the occurrence of *T. sexlineatus* has yet to be verified.

Bidirectional matings between *T*. *septentrionalis* and *T*. *sexlineatus* have been observed several times in the laboratory (Figure 1), and an earlier study using the STRUCTURE analysis of nuclear and mitochondrial DNA data has shown that hybridization and introgression occur in the areas where the two species coexist and become increasingly pronounced as the geographical distance between heterospecific populations decreases (Guo, 2019). Hybridization and introgression may lead to morphological admixture (Milne et al., 1999; Olave et al., 2018; Wu et al., 2020; Yang et al., 2020), and this seems to be also true in this case as revealed by the fact that adult individuals that are morphologically intermediate between the two *Takydromus* species have been found in northern Guangdong where they coexist (Yu, 2014). Specifically, these individuals are similar in body size and shape to *T. septentrionalis* but have a mean number (4.4) of dorsal scales that is much close to that (4) of *T. sexlineatus* but fewer than that (6) of *T. septentrionalis* (Yu, 2014). From the exploited climatic niche hypervolumes, we found a great extent of climatic niche overlap between the two species (Figure 6). This suggests that hybridization and introgression between *T. septentrionalis* and *T. sexlineatus* are likely to take place in any area where climatic conditions are suitable for both species and allow them to coexist.

The upper limit of thermal tolerance (critical thermal maximum, CTMax) does not differ between *T. septentrionalis* and *T. sexlineatus* (~42°C for both species; Qu et al., 2011), whereas the lower limit of thermal tolerance (critical thermal minimum, CTMin) is significantly lower in *T. septentrionalis* (~4°C) than in *T. sexlineatus* (~6°C) (Ji et al., 1996; Zhang & Ji, 2004). In lizards, thermal preference (Tp) is positively associated with CTMax but is independent of CTMin (Qu et al., 2011). This explains why *T. septentrionalis* and *T. sexlineatus* have a similar Tp level (~32°C for both species; Yang et al., 2008). Therefore, as has been reported for other squamate reptiles (Lin & Wiens, 2017; Lin, Zhu, et al., 2019; Lin, Chen, et al., 2019; Stroud et al., 2020), low winter temperature rather than high summer temperature plays a key role in limiting the distribution of the two species farther north. The lower limit of thermal tolerance (CTMin) generally decreases with the increase in latitude and/or altitude in lizards (Qu et al., 2011). It is therefore not surprising that Bio6 contributes the most to the distribution of the two *Takydromus* species and that suitable habitats will shift from their currently known ranges to higher latitudinal and/or altitudinal areas in response to a warming world (Figures 4 and 5). Such geographic (latitudinal and/or altitudinal) trends of climate‐driven changes in distribution have been reported for other taxa including plants and animals (Duan et al., 2016; Frenne et al., 2013; Kraft et al., 2011; Smith et al., 2012; Zhang, Mammola, et al., 2020). Specifically, habitats suitable for *T*. *septentrionalis* in the south will be lost in the predictable future, whereas those suitable for *T*. *sexlineatus* in the area where the two species currently coexist will be preserved or even increase (Figure 5). This may be due to the fact that climate warming adversely affects southern populations of *T*. *septentrionalis* by causing the extinction of these populations or forcing them to move northward (Zhu et al., 2010). Heat loads on animals (i.e., operative temperatures, Te; Bakken, 1992) increase as ambient temperature increases. Terrestrial ectotherms in warm environments with high Te are forced to reduce activity time, as activity is constrained when Te > Tp (Deutsch et al., 2008; Huey et al., 2009, 2010; Sinervo et al., 2010). That climate warming increases the risk of population or species extinctions by constraining the activity time allotted for behavioral thermoregulation, foraging, and other activities has been proved to be true by an empirical experiment on agamid lizards (Wang, Gong, et al., 2017; Wang, Ma, et al., 2017). On the contrary, *T*. *sexlineatus* is likely to benefit from climate warming at least in its northern limit of distribution, where it is sympatric with *T*. *septentrionalis,* but, like other warm‐climate lizards in this area, an even more northerly distribution is constrained by low winter temperature (Du et al., 2011; Ji et al., ,^2002^, ^2006^; Qu et al., 2011; Xu & Ji, 2007).

## 5 CONCLUSIONS

Across the range where the two *Takydromus* species live in sympatry, there are several physical barriers such as Luoxiao Mountains and the Ganjiang River presumed to limit animal migration (Figure 4; Cai et al., 2012). However, the role of these barriers in limiting the migration of terrestrial reptiles including *T*. *septentrionalis* is small or negligible (Cai et al., 2012; Guo et al., 2019; Huang et al., 2007; Lin et al., 2012), thus allowing an inference that introgression and hybridization between *T*. *septentrionalis* and *T*. *sexlineatus* are less likely to be geospatially nonrandom across the range where they are sympatric. Climate warming will not only drive the northward drift of sympatric areas but also increase the size [by up to 58% under the SSP585 (high greenhouse gas emission) scenario] of these areas if nothing is done to limit the emission of greenhouse gases in the future (Figure 5, Table 2). Given the existence of hybridization and introgression between *T. septentrionalis* and *T. sexlineatus* in the field where they coexist, we conclude that climate warming will increase hybridization and introgression between the two species.

## CONFLICT OF INTEREST

The authors have no conflicts of interest to declare.

## AUTHOR CONTRIBUTIONS

**Kun Guo:** Conceptualization (lead); Data curation (lead); Formal analysis (lead); Investigation (equal); Methodology (lead); Project administration (equal); Resources (equal); Software (lead); Writing‐original draft (equal); Writing‐review & editing (equal). **Jun Zhong:** Data curation (equal); Formal analysis (equal); Methodology (equal); Writing‐original draft (equal); Writing‐review & editing (equal). **Fan Xie:** Investigation (equal); Methodology (equal); Software (equal); Writing‐review & editing (equal). **Lin Zhu:** Investigation (equal); Methodology (equal); Software (equal); Writing‐review & editing (equal). **Yan‐Fu Qu:** Data curation (equal); Formal analysis (equal); Supervision (equal); Writing‐original draft (equal); Writing‐review & editing (equal). **Xiang Ji:** Conceptualization (lead); Funding acquisition (lead); Methodology (equal); Project administration (equal); Resources (equal); Software (equal); Supervision (equal); Writing‐original draft (lead); Writing‐review & editing (equal).

## Supporting information

Appendix S1

Figure S1

## Data Availability

The occurrence records used to calibrate and evaluate SDMs are within the article and its supplementary material (Appendix S1).
