# Supplementary figures and images for: Climate warming will increase chances of hybridization and introgression between two Takydromus lizards (Lacertidae)
Source: Ecol Evol. 2021 May 11;11(13):8573–84. doi: 10.1002/ece3.7671 (PMC8258214; doi:10.1002/ece3.7671)

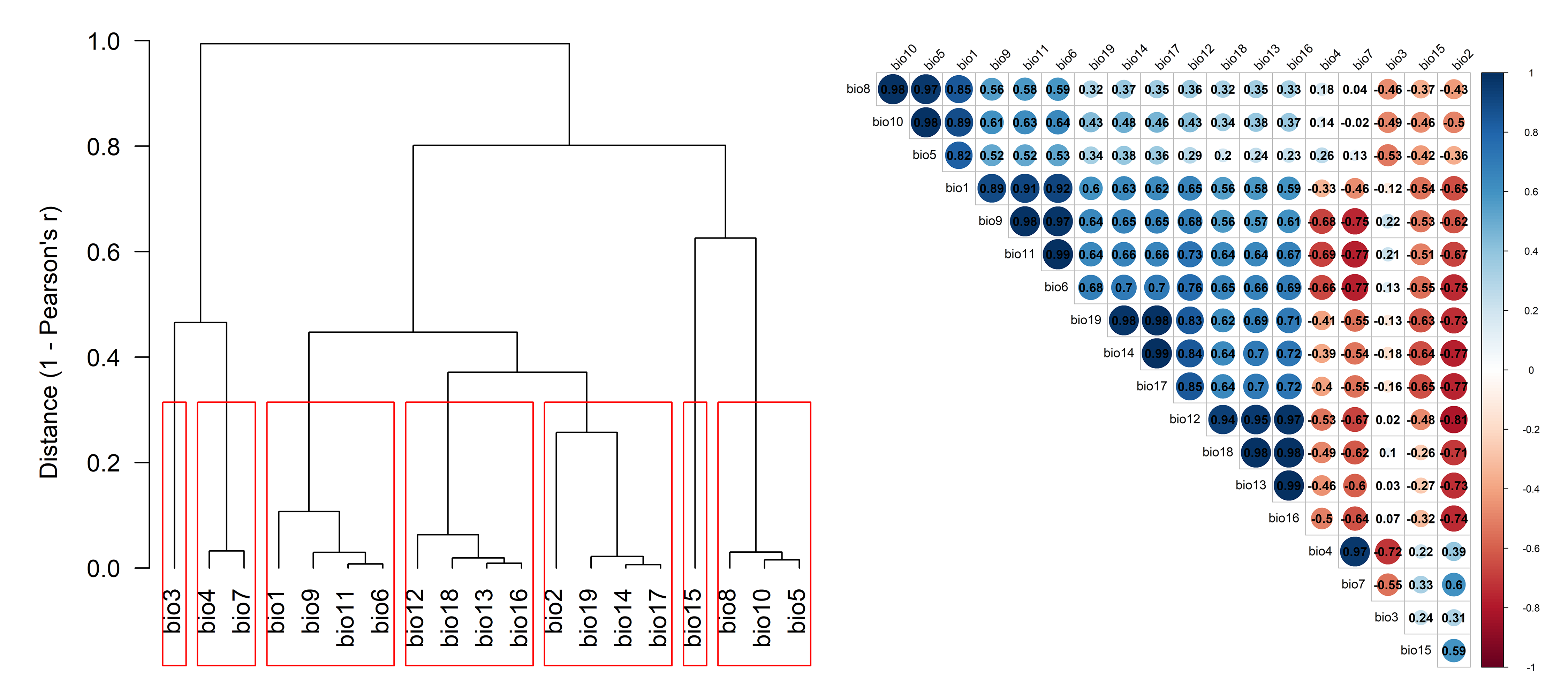

Supplement: Supplementary file 2 — Figure S1 [file ECE3-11-8573-s002.tif]
